# Axotomy Induces Drp1-Dependent Fragmentation of Axonal Mitochondria

**DOI:** 10.3389/fnmol.2021.668670

**Published:** 2021-06-03

**Authors:** Joseph Kedra, Shen Lin, Almudena Pacheco, Gianluca Gallo, George M. Smith

**Affiliations:** ^1^Shriners Hospitals Pediatric Research Center, Lewis Katz School of Medicine, Temple University, Philadelphia, PA, United States; ^2^Department of Anatomy and Cell Biology, Lewis Katz School of Medicine, Temple University, Philadelphia, PA, United States; ^3^Department of Neuroscience, Lewis Katz School of Medicine, Temple University, Philadelphia, PA, United States

**Keywords:** regeneration, mitochondria, mitophagy, corticospinal tract (CST), spinal cord injury

## Abstract

It is well established that CNS axons fail to regenerate, undergo retrograde dieback, and form dystrophic growth cones due to both intrinsic and extrinsic factors. We sought to investigate the role of axonal mitochondria in the axonal response to injury. A viral vector (AAV) containing a mitochondrially targeted fluorescent protein (mitoDsRed) as well as fluorescently tagged LC3 (GFP-LC3), an autophagosomal marker, was injected into the primary motor cortex, to label the corticospinal tract (CST), of adult rats. The axons of the CST were then injured by dorsal column lesion at C4-C5. We found that mitochondria in injured CST axons near the injury site are fragmented and fragmentation of mitochondria persists for 2 weeks before returning to pre-injury lengths. Fragmented mitochondria have consistently been shown to be dysfunctional and detrimental to cellular health. Inhibition of Drp1, the GTPase responsible for mitochondrial fission, using a specific pharmacological inhibitor (mDivi-1) blocked fragmentation. Additionally, it was determined that there is increased mitophagy in CST axons following Spinal cord injury (SCI) based on increased colocalization of mitochondria and LC3. *In vitro* models revealed that mitochondrial divalent ion uptake is necessary for injury-induced mitochondrial fission, as inhibiting the mitochondrial calcium uniporter (MCU) using RU360 prevented injury-induced fission. This phenomenon was also observed *in vivo*. These studies indicate that following the injury, both *in vivo* and *in vitro*, axonal mitochondria undergo increased fission, which may contribute to the lack of regeneration seen in CNS neurons.

## Introduction

Spinal cord injury (SCI) is a severe medical problem with high mortality and long-term morbidity (Eckert and Martin, 2017) for which there is no effective treatment. Following injury, severed axons often undergo dieback/retraction from the site of injury and fail to exhibit subsequent regeneration up to and beyond the injury site. Additionally, many severed axons within the CNS form retraction bulbs at the cut axon tip as opposed to new growth cones (Hill et al., 2001; Hill, 2017). Regenerative failure is due to cell intrinsic (Fawcett and Verhaagen, 2018) and extrinsic factors (Fitch and Silver, 2008). For functional regeneration and repair of injured circuitry to occur, it will be necessary to manipulate both intrinsic and extrinsic factors. Herein, we focus on elucidating the intrinsic factors that impair axon regeneration, specifically focusing on the mitochondrial response to axonal injury in the severed axon.

The mitochondrion has multiple functions in the regulation of cellular physiology by generating ATP, buffering cytosolic calcium, and when dysfunctional, generating excessive reactive oxygen species (ROS; Kasahara and Scorrano, 2014; Brennan-Minnella et al., 2016; Fonteriz et al., 2016; Islam, 2017). In neurons, mitochondria are highly dynamic (Saxton and Hollenbeck, 2012; Schwarz, 2013; Sheng, 2014; Barnhart, 2016). They undergo regulated fission and fusion to adjust their distribution and physiological functions, and if dysfunctional are fragmented by excessive fission before removal by mitophagy. Mitochondria also determine sites of axonal branching and provide important functions in axonal regeneration (Courchet et al., 2013; Spillane et al., 2013; Tao et al., 2014; Cartoni et al., 2016; Han et al., 2016; Winkle et al., 2016; Zhou et al., 2016; Sainath et al., 2017). It is well established that neuronal mitochondria are adversely affected in a variety of neurodegenerative disorders (Abeti and Abramov, 2015; Lane et al., 2015; Zhang et al., 2015; Arun et al., 2016; Bertholet et al., 2016; Krols et al., 2016; Golpich et al., 2017) and following spinal cord injury at the level of whole tissue (Jin et al., 2004; McEwen et al., 2007; Sullivan et al., 2007; Patel et al., 2009, 2010). However, the response of axonal mitochondria to spinal cord injury is yet to be fully understood. The primary features of mitochondrial dysfunction are the fragmentation of mitochondria through repeated bouts of fission into small submicron particles and the concurrent loss of the mitochondrial membrane potential resulting in ATP depletion and generation of ROS. The GTPase Dynamin-related protein 1 (Drp1) is the primary mediator of normal physiological mitochondrial fission, as well as excessive fission leading to fragmentation (Kageyama et al., 2011; Cho et al., 2013; Otera et al., 2013; Sesaki et al., 2014). Mitophagy, a mitochondria selective form of autophagy, serves as a protective mechanism to remove fragmented dysfunctional mitochondria in cells (Karbowski, 2010; Ashrafi and Schwarz, 2013; Balog et al., 2016).

We report that following spinal cord injury, there is a rapid fragmentation of mitochondria in corticospinal tract (CST) axons that persists for up to 3 days following injury before recovery of normal mitochondrial size begins, ultimately attaining sizes similar to uninjured axonal mitochondria 2 weeks after injury. The injury-induced mitochondrial fragmentation requires Drp1 and coincides with an increase in mitophagy that follows a temporal pattern similar to fragmentation. We developed *in vitro* model systems of axonal injury that mimic the *in vivo* response in order to address the injury-induced fission of mitochondria with high spatiotemporal resolution. Using these *in vitro* systems, we find that mitochondrial calcium uptake is required for injury-induced mitochondrial fission, a finding supported by *in vivo* analysis of the role of mitochondria calcium uptake. These insights may pave the way for the development of new therapeutic approaches aimed at the manipulation of axonal mitochondria following injury.

## Materials and Methods

### Animals

All surgical and animal care protocols were approved by the Temple University School of Medicine’s Institutional Animal Care and Use Committee and performed per the National Institutes of Health *Guide for the Care and Use of Laboratory Animals*. Sprague–Dawley rats (65–75 days, 200–224 g; Harlan Laboratories) were housed two per cage, on a 12 h light-dark cycle with food and water provided *ad libitum*. Animals were allowed 7 days of acclimatization prior to any experimental procedure. At the time of surgery, animals ranged in weight from 225 to 250 g. To prevent infection after surgery, animals received an IP injection of 0.5 ml Cefazolin (10 mg/ml). All animals received a subcutaneous injection of 2–3 ml of 0.9% NaCl solution to prevent dehydration following surgery. Analgesia was provided by twice daily oral administration of Rimadyl tablets (1 mg) for 3 days beginning immediately postoperatively.

### Viral Vectors

AAV2-GFP-P2A-mitoDsRed was generated by subcloning the mitoDsRed from the original adenovirus vector (Nasr et al., 2008) into MluI and XhoI sites downstream of the P2A of pAM-CBA-eGFP-P2A. To generate AAV2-eGFPLC3-P2A-mitoDsRed the GFP coding region was removed from AAV2-GFP-P2A-mitoDsRed plasmid and replaced with eGFPLC3 (Addgene: #22405). Purified AAV plasmid was packaged using the helper-free method as reported previously (Ayuso et al., 2010; Liu et al., 2014). In brief, HEK293T cells at 70–80% confluency were transfected with two packaging plasmids, one carrying AAV *rep* and *cap*, the other with AAV helper functions, and the transgene using a polyethylenimine method (PEI; polyethylenimine, linear, MW 25k, Warrington, PA). Three days post transfection, cell supernatant, and lysates were harvested. 40% PEG 8000 was added to precipitate crude virus for 2 h. AAV samples were double-ultracentrifuged in a cesium chloride gradient with isolated viral fractions dialyzed in 0.1 M PBS/0.5% Sorbital overnight (Ayuso et al., 2010; Liu et al., 2014). Purified fractions were added to HEK293T cells to verify function. Quantitative real-time PCR of purified viral fractions was done to determine viral titer. The viral titer of AAV2 was 1.2 × 10^13^ GC/ml.

### Surgical Procedures

#### Cortical Injections

Animals were anesthetized with a ketamine (67 mg/kg)/xylazine (6.7 mg/kg, i.p.) mixture. Their scalps were shaved, and the animals were placed in a stereotaxic head holder. Under aseptic conditions, a small bone flap was cut in the skull to expose the dorsal surface of the primary somatomotor cortex bilaterally. Bone flaps extended from 1 mm anterior to bregma to 1 mm posterior to bregma and from 1 mm from midline laterally to approximately 4.5 mm. Injections into the somatomotor cortex were performed using a beveled glass micropipette pulled to a diameter of 30–40 μm connected to a nanoliter injector (Nanoject, Drummond Scientific). All injections were made using a stereotaxic device or micromanipulator (Narishige International) for precise measurements. All coordinates were determined in relationship to bregma. Bilateral injections of either AAV2-GFP-P2A-mitoDsRed or AAV2-GFPLC3-P2A-mitoDsRed (1.5 μl/injection) were made using the following coordinates: AP axis: +0.5 mm ML axis: +/− 2.5 mm, AP axis: +0.5 mm ML axis: +/− 3.5 mm, AP axis: −0.5 mm ML axis: +/− 3.0 mm, AP axis: −0.5 mm ML axis: +/− 4.0 mm. Coordinates were carefully identified based on the rat brain atlas (Paxinos and Charles, 2007). Following virus injection, all animals were maintained for 4 weeks before cervical spinal cord injury was performed.

#### Corticospinal Tract Severing

Animals were anesthetized with a ketamine (67 mg/kg)/xylazine (6.7 mg/kg, i.p.) mixture. Under aseptic conditions, a 1 cm incision is made through the dorsal skin above vertebra C3-C6. The skin is retracted to expose the underlying muscle. The exposed spinotrapezius muscle is cut along the midline with scissors, then spread with a small Alm retractor. The muscles are freed from the cervical vertebrae with scissors. The first thoracic vertebrae has a very prominent dorsal spinal process for easy identification. A full laminectomy of vertebra C4 and C5 was performed, so as not to damage the spinal cord. Using micro scissors (Vannas Spring Scissors, Fine Science Tools, Inc.) the dorsal column was precisely transected bilaterally (1.5 mm width, 1.1 mm depth). The muscles overlying the spinal cord were loosely sutured together with a 5–0 suture and the wound closed. Animals were allowed to recover at 37°C. Animals were maintained for either 2 h, 8 h, 1 day, 3 days, 1 week, 2 weeks, or 6 weeks following surgery.

For experiments involving mDivi-1 (Cat# 338967-87-6, Sigma-Aldrich) rats had IP injections of either mDivi-1 (50 mg/kg for 250 g rats) or vehicle (10%DMSO/40% PEG300 in saline) 12 h prior to CST severing and immediately after for rats being maintained for 1 day. Rats being maintained for 6 weeks received IP injections 12 h prior to CST severing, immediately after surgery, and once per day for 7 days following the injury.

### Tissue Processing and Histology

At the completion of each experiment, all animals were euthanized by injection of Fatal-Plus (Dearborn, MI, United States) and perfused with saline (0.9% NaCl) followed by 4% paraformaldehyde (PFA) in 0.1 M phosphate buffer (pH 7.5). The brain and spinal cord were promptly dissected. Spinal cord samples were dissected with dorsal roots intact for identification of each spinal level. Samples were post-fixed in 4% PFA overnight at 4°C. Tissue samples were then transferred to a series of graded sucrose (10%, 20%, and 30%) where they remained for the following 24 h or until the brain section sunk to the bottom of the container. A 3 cm section of the spinal cord with the lesion at the center was removed from the whole spinal cord. The spinal cord samples were serially sectioned, sagittally at 30 μm intervals using a Leica CM3050S cryostat. Sections were collected and placed into cryoprotectant solution, then stored at −20°C until processed for histology.

### Immunofluorescence

To amplify the GFP and DsRed signals, sections were permeabilized in 0.3% Triton X-100 with 5% normal goat serum to block non-specific binding sites. Samples were then incubated with chicken-anti-GFP primary antibody (1:1,000; #GFP-1020; Aves Labs Inc., Tigard, OR, United States) and rabbit-anti-DsRed primary antibody (1:500; #632496; Takara Bio USA, Inc., Mountain View, CA, United States) overnight at 4°C. The next day, samples were incubated with donkey-anti-chicken-AlexaFluor 488 sary antibody (1:400, Jackson ImmunoResearch Laboratories, Inc., West Grove, PA, United States) and goat-anti-rabbit-AlexaFluor 594 sary antibody (1:400, Jackson ImmunoResearch Laboratories, Inc., West Grove, PA, United States) then mounted on glass slides.

### Tissue Section Imaging

Imaging was performed using AxioVision software (Carl Zeiss Microscopy, Thornwood, NY, United States). A 100× /1.3 N/A objective with a ~300 nm focal plane was used to generate Z-stacks using 250 nm Z-intervals through 30 μm of tissue sections. In order to maximize the power of the morphological analysis, 1 × 1 camera binning was used to generate the highest spatial resolution in the images. Max-intensity 2D projections were then generated using sets of sections from the Z-stack that contained mitochondria.

### Culturing of Primary Neurons

Chicken dorsal root ganglia (DRG) were dissected at embryonic day 7 (E7) or day 14 (E14; SPF eggs obtained from Charles River Laboratories). At this developmental stage, it is not possible to determine the sex, and both sexes were used at presumably a 50/50 ratio. Whole explants were cultured (3–4 explants/dish), or the DRG neurons were dissociated and transfected (as explained below; 1.5 explants/dish). The culturing substrata were previously coated with polylysine [Sigma; Catalog number (Cat#) P9011; 100 μg/ml in borate buffer], for 4 h and following 3× washing with phosphate-buffered saline (PBS) with 25 μg/ml laminin (Life Technologies Cat# 23017-015) in PBS overnight, all incubations were performed at 39°C. Explants and dissociated neurons were cultured in a defined F12H medium (Gibco; Cat#21700075) supplemented with NGF (20 ng/ml). For live imaging experiments, explants or dissociated neurons were plated in glass-bottom dishes.

For the preparation of dissociated neurons, sensory ganglia were incubated in Ca^2+^–Mg^2+^-free PBS (CMF-PBS), for 10 min at 37°C. Ganglia were then spun down for 1 min, and the supernatant was removed. Ganglia were then treated with 0.25% trypsin (Fisher Scientific Cat# MT25005CI), for 10 min at 37°C and spun down for 1 min. Ganglia were then pipette triturated 30 times in F12HS10 media (F12H medium supplemented with 10% fetal bovine serum: Fisher; Cat#MT350111CV) and then spun down for 4 min. The supernatant was removed and cells were transfected as described below.

Rat hippocampal neurons were dissociated from dissected E18 rat hippocampi as previously described (Thomas et al., 2012). Neurons were cultured in Neurobasal media supplemented with B27 (Thermo Fischer Scientific) in glass-bottomed dishes coated with polylysine [Sigma; Catalog number (Cat#) P9011; 100 μg/ml in borate buffer] and laminin (Life Technologies Cat# 23017-015; 25 μg/ml) as described above.

Adult rat DRG dissociated cultures were prepared as described previously (Pacheco et al., 2020).

### Transfection

For transfection of plasmids into neurons, 40 chicken DRGs were dissociated as described above, and after F12HS10 was removed neurons were suspended in 100 μl nucleofector solution (Lonza Cat# VPG-1002) and gently resuspended through trituration. The DRG cell suspension was transferred to a nucleofector cuvette containing 10 μg of Plasmid DNA and electroporated using an Amaxa Nucleoporator (program G-13). The electroporated solution was then immediately transferred to a tube containing F12H media as described above prior to plating.

### Labeling of Mitochondria *In vitro*

For labeling mitochondria to determine mitochondrial fission rates, neurons were transfected with pDsRed2-Mito (Clontech Cat# 632421) as explained above. To measure mitochondrial length, mitochondria were labeled with mitochondria targeted dyes, which were prepared according to the manufacturer’s directions. Labeling was performed through incubation for 30 min with MitoTracker green (50 nM, Molecular Probes, Cat# M7514) or Mitoview 633 (20 nM, Biotium^®^, Heyward, USA; Cat# 70055-T). Following labeling, the dye-containing medium was removed and cultures were washed three times with culturing medium not containing any dye. Imaging was performed at least 30 min after the dye was removed to allow cultures to acclimate.

### *In vitro* Axonal Severing

For experiments in which DRG explants were used, explants were plated in glass-bottom dishes as described above and cultured for 48 h. Dishes were placed on the microscope stage for 10 min prior to severing. Axons were severed by dragging a No. 11 scalpel blade across the axon perpendicular to its direction of growth.

For experiments in which dissociated, transfected neurons were used, neurons were cultured in glass-bottom dishes as described above for 24 h, with the exception of neurons transfected with GCaMP6, which were cultured for 72 h (the time at which expression of GCaMP6 was optimal). Dishes were placed on the stage 10 min prior to severing. Severing was performed as previously described (Gallo, 2004). Briefly, only axons that exhibited a “healthy” uniform caliber axon were used in single-axon-severing experiments. The severing of axons was performed approximately 200 μm from the axon tip. For severing, borosilicate glass capillaries (AM Systems, Carlsborg WA; catalog # 625500) were pulled to fine tips using a Pul-1 micropipette puller (World Precision Instruments, Sarasota FL). Capillary needles were mounted on a mechanical micromanipulator (Leica Inc., Bannockburn, IL). The tip was then brought into contact with the substratum and axons severed by maintaining the position of the tip steady while moving the stage relative to the tip. The stage was moved manually in a continuous swift motion resulting in the uniform severing of axons.

### Live Imaging

Neurons were imaged using a Zeiss 200 M microscope equipped with an Orca-ER camera (Hamamatsu) in series with a PC workstation running Zeiss Axiovision software for image acquisition and analysis. Cultures were placed on a heated microscope stage (Zeiss temperable insert P with objective heater) for 10 min at a constant 39°C before and during imaging. Imaging was performed using a Zeiss Pan-Neofluar 100_objective (1.3 N.A.), 2 × 2 camera binning, and minimal light exposure. For the quantification of mitochondria length, mitochondria labeled with MitoTracker green (Chicken sensory neurons) or Mitoview 633 (rat hippocampal neurons), were imaged at 30 min intervals over a 180 min timeframe. For mitochondrial fission and attempted fission rate quantification, neurons were co-transfected with pDsRed2-Mito and either pEYFP-C1-Drp1 or pEYFP-C1-Drp1K38A [previously generated in our lab (Armijo-Weingart et al., 2019)]. Neurons were then imaged for 250 frames with a 3 s interframe interval. For analysis of changes in cytosolic and mitochondrial calcium, neurons were transfected with either GCaMP6 or mitoGCaMP2. Neurons were then imaged for 16 frames with a 1 min interframe interval.

### Determination of Mitochondria Length

Imaging was performed as described in the tissue imaging and live imaging sections above. The length of mitochondria in the axon was measured using ImageJ software. Mitochondria length was determined by a line, segmented if the mitochondrion was not strictly linear, running from one end to the other of the mitochondrion. Occasionally, mitochondria in axons overlapped as clearly evidenced by the additive signal of their fluorescence in the region of overlap. Overlapping mitochondria were excluded from the analysis to ensure only mitochondria with clearly defined ends were used for quantification.

### Determination of Rates of Fission and Fusion From Live Imaging Time-Lapse Videos

Instances of fission were determined manually by the user through frame-by-frame analysis. Using the settings described in the live imaging section each pixel in the resulting images has a radius of 0.12 μm. A completed fission event was defined as when the fluorescence intensity of the mitochondrion at the site of apparent fission had decreased to the background level of the axonal segment not containing mitochondria and the two resultant mitochondria were separated by over 5 pixels representing 0.6 μm. Attempted fission events were defined as mitochondria undergoing constrictions but not continuing into a full fission event, as the constriction sites returned to exhibit the prior width and intensity.

### GCaMP6 and mitoGCaMP2 Analysis

All analyses were performed using Zeiss Axiovision software. For GCaMP6, regions of interest (ROIs) were drawn around the cut axon tip and 50 μm proximal to the cut. For mitoGCaMP2, ROIs were drawn around axonal mitochondria proximal to the cut site. Image acquisition was performed using excitation filters of 405 nm and 488 nm (Need specifications on Filters). Fluorescence time courses were computed as the mean pixel intensity of all the pixels within the ROI. At each timepoint, fluorescence intensity for both excitation filters (*F*_405_ and *F*_488_) was calculated as mean fluorescence within the ROI, *F*, relative to the background *F*_0_ (*F* − *F*_0_). Data is reported as a ratio of *F*_488_/*F*_405_.

### Statistical Analysis

All data were analyzed using Instat software (GraphPad Software Inc.). The software determines the normalcy of data sets using the Kolmogorov-Smirnov test. All data sets were determined to be non-normally distributed, therefore non-parametric analysis was used (Mann-Whitney test). For multiple comparison tests within experimental designs, non-parametric Dunn’s *post hoc* tests were used. For data sets representing categorical data falling into bins the Fischer’s exact test was used on the raw data, although the data are expressed as percentages for ease of appreciation. One or two tailed *p* values are reported based on whether the hypothesis did or did not dictate the directionality of the expected change in mean or median, respectively. All graphs were generated using Prism (GraphPad Software Inc.). Sample sizes and qualitative statistical presentation are denoted in figure legends or figures.

## Results

### Spinal Cord Injury Decreases the Length of Axonal Mitochondria

**Figure 1:**
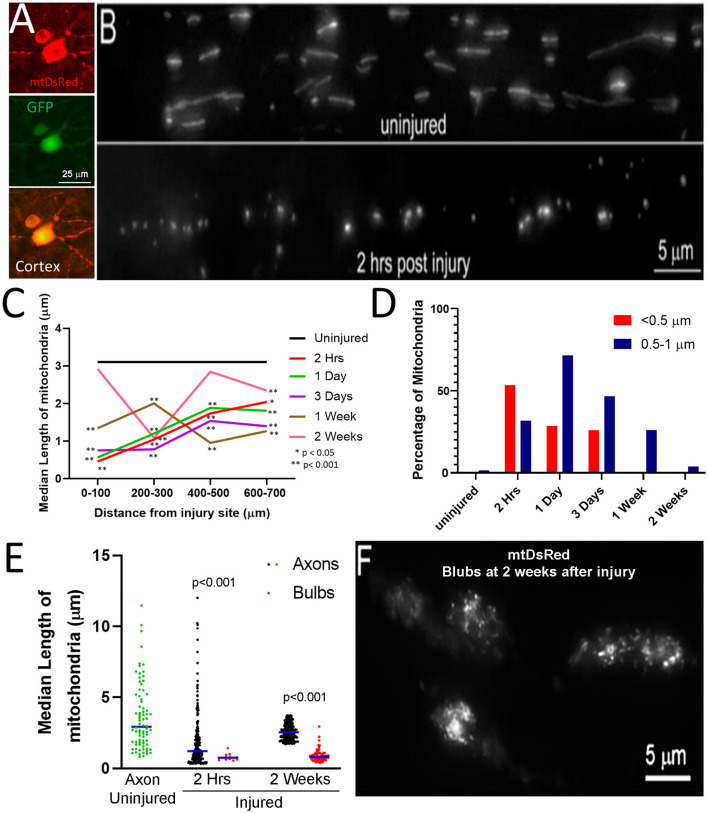
Fragmentation of axonal mitochondria following injury. **(A)** Examples of layer V neurons expressing mtDsRed and GFP. **(B)** Examples of mtDsRed labeled mitochondria in CST axons at C4–5 in the uninjured control and at 2 h post-injury. **(C)** Graph of the median mitochondrial length as a function of time post-injury and distance from injury (Dunn multiple comparison tests). *n* = 3 rats per group. *p < 0.05, **p < 0.01. **(D)** Graph of the percentage of mitochondria in the <0.5 and 0.5–1. 0 μm bins as a function of time at 0–100 μm from the injury site. **(E)** Graph of the median mitochondrial lengths in retraction bulbs and axons (Mann-Whitney test). **(F)** Example of fragmented mitochondria in bulbs at 2 weeks after injury.

To assess axonal mitochondrial morphology following spinal cord injury, the mitochondria of CST neurons of adult Sprague–Dawley rats were labeled with mitochondrially targeted DsRed (mtDsRed) 4 weeks before performing dorsal column lesions at the C4-C5 level. Labeling was achieved by injecting adeno-associated virus co-expressing GFP and mitoDsRed (mitoDsRed/GFP/AAV) in the forelimb and hindlimb somatomotor cortex (Figures 1A,B). The mitochondria in non-injured axons at the C4-C5 level had a median length of 3.15 μm (Figure 1C). Mitochondria in C4-C5 CST axons severed following dorsal column lesion were analyzed in 100 μm bins from the site of injury as a function of time following injury. At 2 h following injury, mitochondria closest to the site of injury exhibited decreased length, while mitochondria more proximal along the injured axon maintained longer lengths although still decreased relative to controls (Figure 1C). Very small mitochondria in the vicinity of the injury site persisted for up to 3 days following injury (Figure 1C). Recovery of median mitochondrial length began by 1 week after injury with normal lengths almost fully restored by 2 weeks (Figure 1C; see Supplementary Figure 1 for data point distributions of the medians presented in Figure 1C).

Mitochondria in the <0.5 μm range are considered dysfunctional (Reddy, 2014; Galluzzi et al., 2016; Hu et al., 2017). In uninjured axons, less than 0.5% of mitochondria were shorter than 0.5 μm and only 4.4% of mitochondria fell within the 0.5–1.0 μm range. Herein, mitochondria with length <0.5 μm are defined as fragments, while mitochondria 0.5–1.0 μm are considered to be in the low normal range for length. By 2 h following injury, the percentage of mitochondria in the submicron range dramatically increased resulting in 53% of mitochondria displaying lengths <0.5 μm and 32% with length between 0.5 and 1.0 μm (Figure 1D). The presence of increased mitochondrial fragments persisted until 3 days following injury before returning to non-injury control levels by 1-week post-injury (Figure 1D). Fragmented mitochondria were identified throughout the distal region of severed axons and the axon tips. However, the length of mitochondria within retraction bulbs, defined as the distal end of the axon exhibiting a bulbous rounded morphology, relative to those in non-retraction bulb segments of axons, continued to exhibit sub-μm fragmentation up to at least 2 weeks (Figures 1E,F).

### Fragmentation of Axonal Mitochondria Is Associated With Increased Colocalization of Mitochondria With Mitophagy Markers Following Spinal Cord Injury

**Figure 2:**
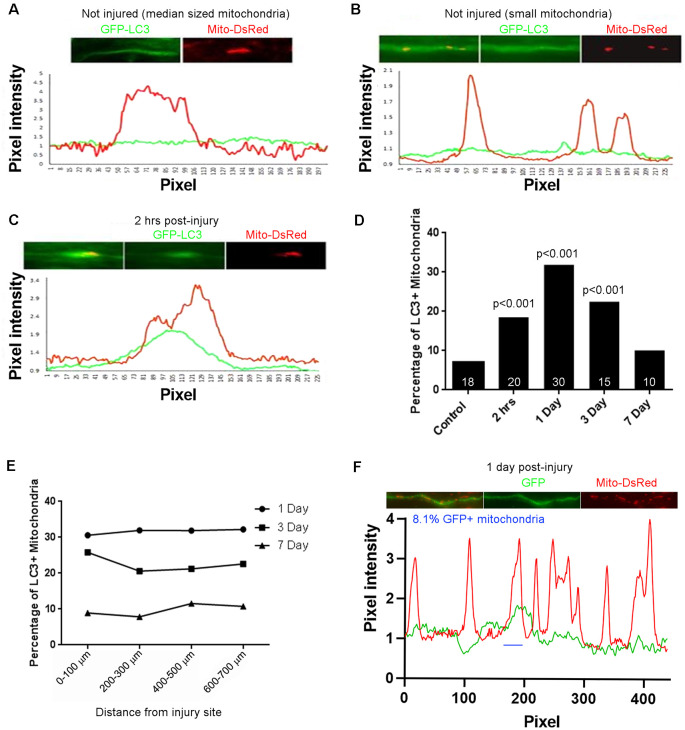
Injury-induced accumulation of LC3 at sites populated by mitochondria. **(A)** Example of LC3 distribution in uninjured axons relative to mitochondria. Line scan shows no increase in LC3 at the mitochondrion. For both traces, values are normalized to the mean intensity in regions not containing mitochondria. **(B)** As in **(A)** but showing three uninjured small mitochondria not showing LC3 accumulation. **(C)** Two hours after injury, LC3 is observed associated with mitochondria. Positive LC3 accumulation was defined as a maximal increase greater than 15% from the mean baseline in the axon segment defined by the mitochondrion. **(D)** Percentage of LC3+ mitochondria as a function of time post-injury. n = axons shown in bars. **(E)** Graph of percentage of LC3+ mitochondria as a function of distance from the site of injury. **(F)** Analysis of colocalization of GFP intensity with mitochondria positioning along axons. Using the same criteria as for GFP-LC3, at 1 day after injury reflective of when the percentage of GFP-LC3+ mitochondria peaks, only 8.1% of mitochondria are GFP+ (one case denoted by blue line). *n* = 3 rats per group.

Fragmented dysfunctional mitochondria are removed through mitophagy1 (Ashrafi and Schwarz, 2013). To determine if the mitochondrial fragmentation along CST axons observed after spinal cord injury correlates with increased mitophagy, we examined the association of mitochondria with the autophagocytic marker LC3 (Maday, 2016; Maday and Holzbaur, 2016). Adult Sprague-Dawley rats were injected with AAV co-expressing GFP-LC3 and mitoDsRed into the somatomotor cortex as described above. Four weeks after injections, the rats received a dorsal column lesion at the C4-C5 level to sever labeled CST axons. Line scans of fluorescent intensities from randomly selected axons were analyzed to measure the degree of LC3 association with mitochondria. Within the C4-C5 level of non-injured rats, GFP-LC3 florescence (Figures 2A,B, green line) was mostly uniformly distributed with few regions showing increased pixel intensity associated with DsRed labeled mitochondria (Figure 2A, red line). Prior to the injury, we observed that only 7.1% of axonal mitochondria were associated with localized increases in LC3. At 2 h after injury, 18.5% of mitochondria were associated with localized increases in the levels of LC3 (Figures 2C,F; hereafter referred to as LC3+ mitochondria). The increase in LC3+ mitochondria reached its peak 1 day after injury (31.8% LC3+ mitochondria) but continued until 3 days after injury (22.5% LC3+ mitochondria) before returning to non-injury control levels 7 days after injury (10.1% LC3+ mitochondria; Figure 2D). The percentage of LC3+ mitochondria was not dependent on the distance from the injury site, as the percentage of LC3+ mitochondria was the same at all distances from the injury site at each time point (Figure 2E). These observations indicate that there is an increase in mitophagy that correlates with mitochondrial fragmentation up to 3 days following spinal cord injury. To determine if the association of GFP-LC3 accumulation along axon with mitochondria might be due to volumetric or non-specific changes, we considered the association of GFP accumulation with mitochondria. For this analysis we focused on 1 day after the injury as at this time point reflects the peak value of the percentage of LC3+ mitochondria (Figure 2D). Using the same criteria and analysis as for GFP-LC3, at 1 day after injury 8.1% of mitochondria (*n* = 20 axons, 148 mitochondria) were found associated with accumulation of GFP. These data indicate that the accumulation of GFP-LC3 noted in the injury conditions is not non-specific.

### Inhibition of Drp1-Mediated Fission Prevents Injury-Induced Fragmentation of Axonal Mitochondria

**Figure 3:**
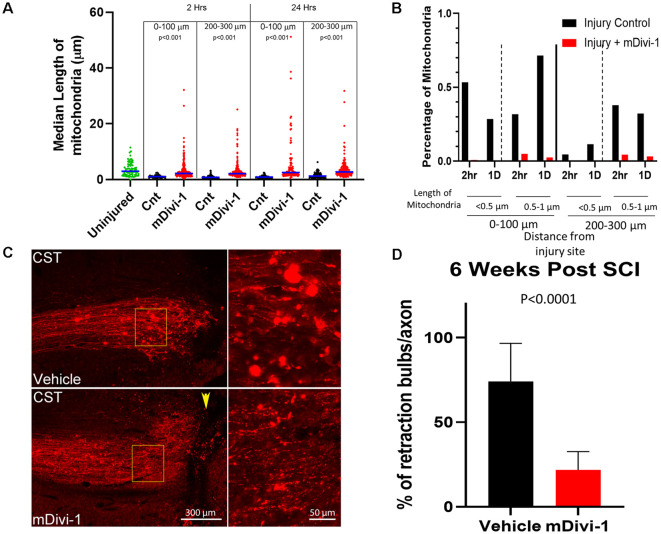
mDivi1 blocks mitochondrial fragmentation and reduces the number of retraction bulbs. **(A)** Graph of median mitochondrial lengths with and without mDivi-1 treatment (Dunn Multiple Comparison test) *n* = 3 rats per group Statistical testing done in comparison to time-matched vehicle controls. **(B)** Percentage of mitochondria in the <0.5 (fragmented) and 0.5–1 μm ranges with and without mDivi-1 treatment. **(C)** Examples of CST axons containing mitoDsRed labeled mitochondria adjacent to the injury site. In inset: note that the control exhibits numerous bright spherical retraction bulbs further from the lesion site and adjacent to the cavity. However, with mDivi-1 treatment, no cavities formed, axon did not appear to retract from lesion site, and the number of retraction bulbs greatly decreased. **(D)** Mean % of axons with retraction bulbs. *n* = 9 rats for each group. The data were analyzed using a Fisher’s test on the raw aggregated categorical data within the group.

We next sought to assess the role of Drp1 mediated fission in the injury-induced mitochondrial shortening observed *in vivo*. mDivi-1 is a cell and blood-brain barrier permeable pharmacological inhibitor of Drp1 that blocks mitochondrial fission both *in vitro* and *in vivo* (Lackner and Nunnari, 2010; Li et al., 2016; Smith and Gallo, 2017). Adult Sprague-Dawley rats were injected with mitoDsRed/AAV in the somatomotor cortex 4 weeks prior to CST severing at the C4-C5 level. mDivi-1 or vehicle was delivered *via* IP injections at 12 h before injury, and again immediately post-injury for the 1 day survival group, similar to published *in vivo* protocols (He et al., 2016). Mitochondria were then analyzed at 2 h and 1-day post-injury in the distal 0–300 μm of axons. mDivi-1 treatment decreased injury-induced mitochondrial fragmentation at 2 h and 1 day after injury. At 2 h, mDivi-1 treatment resulted in mitochondria with intermediate lengths, ranging between the non-injury and injury conditions (Figure 3A). However, the 1-day treatment group exhibited mitochondrial lengths not significantly different than non-injured controls (Figure 3A). Analysis of the percentage of mitochondria in the fragmented range (<0.5 μm) and lower end of the normal range (0.5–1 μm) revealed that mDivi-1 treatment prevented fragmentation at both 2 h and 1 day (Figure 3B).

### The Drp-1 Fission Inhibitor mDivi-1 Reduces the Number of Retraction Bulbs on CST Axons

The fragmentation of axonal mitochondria was rapid and persistent in retraction bulbs even at longer times after injury. In order to determine whether mitochondria fragmentation could be a component of the mechanism that generates or supports retraction bulbs, the effects of mDivi-1 treatment on bulbs at the end of injured axons was examined. Adult Sprague-Dawley rats were injected with mtDsRed/GFP/AAV into the somatomotor cortex 4 weeks prior to the severing of CST axons at the C4-C5 level. The rats received IP injections of either mDivi-1 or vehicle the day before injury and once per day for 7 days after injury. Six weeks following injury, the animals were euthanized, and the spinal cord segments were analyzed. Vehicle injected controls showed cavitation at the injury site, axonal retraction, and numerous retraction bulbs at the distal tips of injured CST axons (Figure 3C). Consistent with a prior report (Li et al., 2016), animals treated with mDivi-1 showed a decreased lesion volume with lesioned axons adjacent the lesion site. In addition, there was a 73% decrease in the number of retraction bulbs observed at axon tips (Figure 3D).

### Development of *In vitro* Model Systems to Study the Fission Response of Mitochondria to Axonal Injury

**Figure 4:**
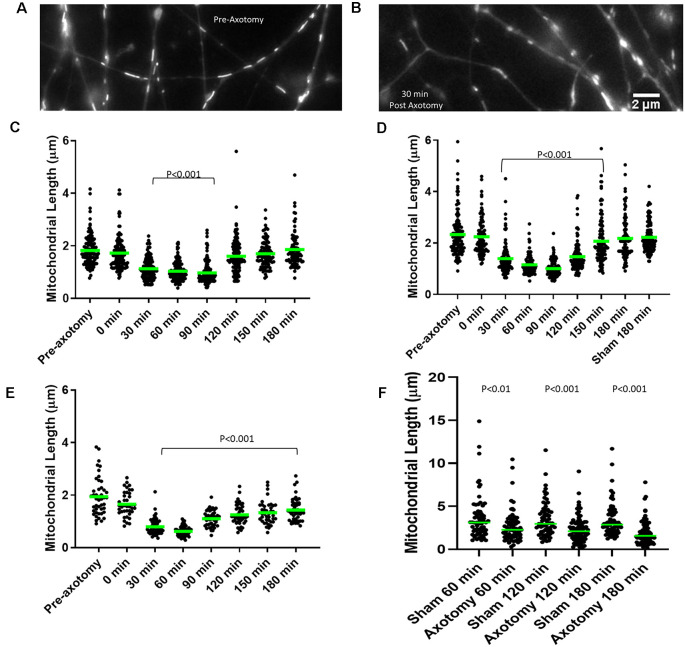
Axonal injury results in mitochondrial shortening *in vitro*. **(A)** Example of mitochondria stained with MitoTracker green in E14 chicken dorsal root ganglia (DRG) axons prior to axotomy. **(B)** Example of mitochondria in E14 chicken DRG axons 30 min following axotomy. **(C)** Median mitochondrial length in embryonic day 7 (E7) chicken DRG axons at 30 min intervals following axotomy. Dots represent individual mitochondria. *n* = 15 axons. **(D)** Median mitochondrial length in E14 chicken DRG axons at 30 min intervals following axotomy. *n* = 15 axons. **(E)** Median mitochondrial length in E18 rat hippocampal axons at 30 min intervals following axotomy. *n* = 10 axons. **(F)** Median mitochondrial length in adult rat DRG axons at 60 min intervals following axotomy *n* ≥ 9 axons per group. All statistical analyses done with Dunn’s multiple comparison tests.

In order to perform mechanistic studies not amenable to *in vivo* examination and to determine whether the response of axonal mitochondria to axon severing may be cell-intrinsic within the cut end of axons, we sought to set up *in vitro* model systems for examining mitochondrial response to axonal injury. We investigated mitochondrial morphology after *in vitro* axonal injury using neurons from two species and representing early embryonic, late embryonic, and adult stages: (1) Embryonic day (E)7 and E14 chicken DRG explants, reflective of developmental stages when sensory axons are undergoing extension and have reached their proximal targets respectively (Figures 4A–D). (2) E18 rat dissociated hippocampal neurons (Figure 4E) and (3) adult rat DRG dissociated neurons (Figure 4F).

Mitochondria within embryonic chicken sensory axons were labeled with MitoTracker Green dye and axons were severed using a scalpel blade after 48 h in culture when axons attained lengths between 1–1.5 mm. The mitochondrial length was monitored using live imaging. Prior to scalpel axotomy, axonal mitochondria showed median lengths of 1.63 μm and 2.41 μm in E7 and E14 neurons, respectively (Figures 4A–D), consistent with the prior demonstration of increased length in E14 neurons relative to E7 neurons (Armijo-Weingart et al., 2019). Following axotomy, both E7 and E14 axons showed a significant reduction in mitochondrial length 30 min after injury. Mitochondrial length began to recover between 120 min and 150 min post-injury before returning to pre-injury levels 180 min following injury (Figures 4C,D). The recovery from injury was faster in E7 (120 min) than E14 axons (180 min). E18 hippocampal neurons were cultured for 3 days, the time point at which axons attained lengths between 300 and 400 μm (stage III of *in vitro* development). Axonal mitochondria were labeled with Mitoview 633. Mitochondrial length decreased 30 min after injury and did not recover fully until at least 180 min (Figure 4E). Similar analysis using adult rat DRG neurons with mitochondria labeled using MitoTracker Green showed that lengths were decreased one hour after injury and the decrease in length persisted until sometime after 180 min (Figure 4F).

**Figure 5:**
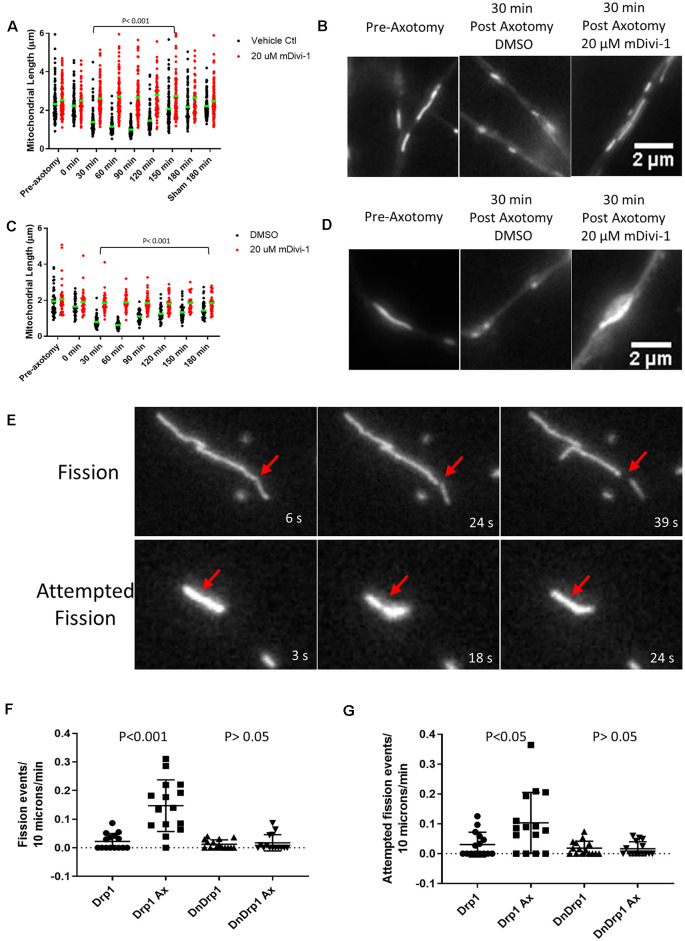
Injury-induced mitochondrial fission is Drp1 dependent *in vitro*. **(A)** Median mitochondrial length in E14 chicken DRG axons with or without mDivi-1. Dots represent individual mitochondria (Dunn’s multiple comparison tests). *n* = 15 axons for each group. **(B)** Examples of MitoTracker green stained mitochondria in E14 chicken DRG axons. **(C)** Median mitochondrial length in E18 rat hippocampal axons with or without mDivi-1. Dots represent individual mitochondria (Dunn’s multiple comparison tests). *n* = 10 axons for each group. **(D)** Examples of Mitoview633 stained mitochondria in E18 rat hippocampal axons. **(E)** Example of mitochondria labeled with mitoDsRed undergoing fission or attempted fission following axonal injury. At 18 s a constriction is apparent at the site denoted by the arrow but by 24 s the constriction is no longer detectable, and the mitochondrion has returned to its prior morphology. **(F)** Graph of fission events per 10 μm of mitochondrial length per minute in E14 chicken DRG axons transfected with either YFP-Drp1 or YFP-Drp1K38A (DNDrp1) following injury (Dunn’s multiple comparison tests). *n* = 15 axons. **(G)** Graph of attempted fission events per 10 μm of mitochondrial length per minute in E14 chicken DRG axons transfected with either YFP-Drp1 or YFP-Drp1K38A (DNDrp1) following injury (Dunn’s multiple comparison tests). *n* = 15 axons.

We next tested whether injury-induced mitochondrial shortening in sensory axons is Drp1 dependent *in vitro*. E14 chicken DRG explants were incubated with either DMSO (control) or 20 μM mDivi-1 for 30 min prior to scalpel blade axotomy. Mitochondria within sensory axons were labeled with MitoTracker green. Following axotomy mitochondrial size was monitored for 180 min. As with untreated neurons (Figure 4D), DMSO treated neurons demonstrated prominent mitochondrial shortening 30 min after injury before gradually returning to pre-injury lengths by 180 min. Neurons treated with mDivi-1 did not show mitochondrial shortening at any time point following axotomy (Figures 5A,B). Additionally, we tested whether injury-induced mitochondrial shortening in hippocampal neurons is Drp1 dependent *in vitro*. Axonal mitochondria length was measured in E18 hippocampal neurons (as in Figure 4E) treated with either DMSO or 20 μM mDivi-1 30 min before severing using a micro-needle as previously described (Gallo, 2004). The mitochondrial length was monitored using live imaging. Prior to axotomy, DMSO treated neurons displayed axonal mitochondria with a median length of 1.86 μm. The median mitochondrial length was significantly reduced at 30 min after axotomy. By 180 min following injury, median mitochondrial length was still significantly smaller than before axotomy but was trending toward recovery of pre-axotomy length (Figures 5C,D). Neurons treated with 20 μM mDivi-1 showed no change in median mitochondrial length over the 180-min monitoring period (Figure 5C). These observations indicate that injury-induced mitochondrial shortening *in vitro* is a Drp1 dependent process.

To further test the hypothesis that mitochondria shortening is due to Drp1 mediated fission, we expressed a dominant-negative Drp1 mutant (Drp1K38A) that lacks GTPase activity and is, therefore, unable to carry out mitochondrial fission (Pitts et al., 1999; Barsoum et al., 2006). E14 chicken DRGs were transfected with either YFP-Drp1 (control) and mitoDsRed or YFP-Drp1K38A and mitoDsRed. Single axon axotomy was performed using a micro-needle as done previously (Gallo, 2004). Because transfection with Drp1K38A allows for unopposed mitochondrial fusion, mitochondria become elongated (20–30 μm or larger) in sensory neurons by 24 h of expression (Armijo-Weingart et al., 2019). Thus, comparison of mitochondrial length after axotomy between control and Drp1K38A expressing neurons is not a reasonable analysis. We, therefore, measured the number of fission events and attempted fission events per 10 microns of mitochondria per min for each group monitored over a 15-min period. As previously described (Armijo-Weingart et al., 2019), a fission event was defined as when the fluorescence intensity of the mitochondrion at the site of apparent fission had decreased to the background level of the axonal segment not containing mitochondria and the two resultant mitochondria were separated by over 5 pixels representing 0.6 μm (Figure 5E). Attempted fission events were defined as mitochondria that underwent apparent constrictions but did not continue into a full fission event, as the constriction sites returned to exhibit the prior width and intensity (Figure 5E). It should also be noted that overexpression of WT Drp1 does not increase mitochondrial fission events at baseline (Armijo-Weingart et al., 2019). Axotomy resulted in increased fission and attempted fission events in control YFP-Drp1 expressing neurons (Figures 5F,G). The increases in fission and attempted fission events in response to axotomy were abolished by expression of YFP-Drp1K38A (Figures 5F,G).

### Injury-Induced Mitochondrial Fission Requires the Mitochondrial Calcium Uniporter

**Figure 6:**
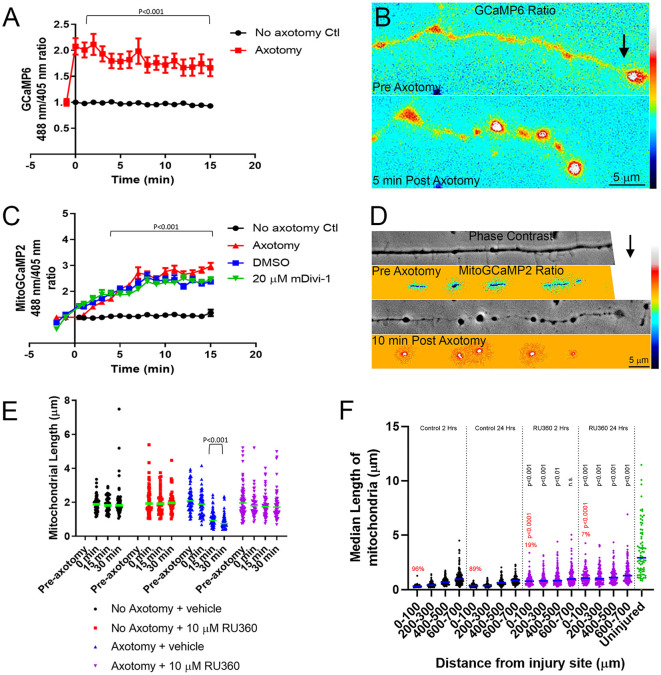
Injury-induced mitochondrial fission requires mitochondrial calcium uptake both *in vitro* and *in vivo*. **(A)** Graph of cytosolic GCaMP6 fluorescence ratio (488 nm/405 nm) in E7 chicken DRG axons following axotomy (Dunn Multiple Comparison test). *n* = 10 axons per group. **(B)** Ratiometric false-colored images of the GCaMP6 signal in a representative axon pre and 5 min post axotomy. The black arrow denotes the location of the axotomy. As reflected by the ratiometric measurements by 5 min the GCaMP6 signal is elevated, particularly in axonal swellings that form after axotomy. **(C)** Graph of mitoGCaMP2 fluorescence ratio (488 nm/405 nm) in E7 chicken DRG axons following axotomy with or without mDivi-1 (Dunn Multiple Comparison test). *n* = 10 axons per group. **(D)** Ratiometric false-colored images of the mitoGCaMP2 signal in a representative axon pre and 5 min post axotomy. As in panel **(B)** the arrow denotes the location of axotomy. In this example, the field of view containing the axon was shifted leftward while the axotomy was occurring and is not visible in the preimages. As shown quantitatively in panel **(C)** the ratio in mitochondria is increased by 10 min post-injury. **(E)** Graph of median mitochondrial length in E7 chicken DRG axons with or without axotomy and with or without RU360 (Dunn Multiple Comparison test). *n* = 10 axons per group. **(F)** Graph of median mitochondrial length in adult rat CST axons following the injury at the C4-C5 level with or without RU360 (Dunn Multiple Comparison test). *n* = 3 rats per group. Statistical testing done in comparison to time-matched vehicle controls. All groups are different from the uninjured at *p* < 0.0001. The percentage of fragmented (<0.5 micron) mitochondria in the 0–100 micron binds is shown in red. Fisher exact tests between time-matched control and 10 mM RU360 treatment groups are shown in red.

Following axotomy, there is an influx of calcium into the axon, which is important in the formation of either a retraction bulb or a new growth cone (Bradke et al., 2012). We first transfected E7 chicken DRG neurons with GCaMP6, a cytosolic, ratiometric calcium-sensing protein (Chen et al., 2013). Individual axons were severed using a micro-needle, as above, and calcium changes were monitored over a 15-min period with live imaging. As expected axotomy caused a sustained influx of calcium resulting in a two-fold increase in GCaMP6 fluorescence (Figures 6A,B). To determine whether intra-mitochondrial calcium levels are increased following axotomy, E7 chicken DRG neurons were transfected with a mitochondrially tagged calcium-sensing protein, mitoGCaMP2. We monitored mitochondrial calcium levels for 15 min following axotomy, observing a steady increase in mitochondrial calcium culminating in a three-fold increase in mitoGCaMP2 fluorescence at 15 min post axotomy (Figures 6C,D). To determine if this increase in mitochondrial calcium was dependent on injury-induced fission, we pretreated neurons with either DMSO or 20 μM mDivi-1 as above. Blocking fission following axotomy had no effect on mitochondrial calcium demonstrating that this was not a fission-dependent process (Figure 6C). Given the increase in cytosolic calcium after axotomy, we next addressed if injury-induced mitochondrial fission may be dependent on an increase in mitochondrial calcium. Calcium is taken up into mitochondria through the mitochondrial calcium uniporter (MCU) present on the mitochondrial inner membrane (Gunter and Gunter, 1994). RU360 is an established inhibitor of calcium fluxes through MCU (Matlib et al., 1998; Zhou et al., 1998; Vanderluit et al., 2003). E7 chicken DRG explants were treated with either 10 μM RU360 or vehicle and mitochondria were labeled with MitoTracker Green 30 min prior to scalpel axotomy. Following axotomy, mitochondrial size was monitored for 30 min using live imaging. Application of RU360 had no effect on mitochondrial size in the absence of axotomy during the 30-min imaging period (Figure 6E). At 30 min after axotomy, vehicle treated neurons displayed a significantly reduced median mitochondrial length when compared to mitochondrial length prior to axotomy, while neurons treated with 10 μM RU360 displayed no change in median mitochondrial length (Figure 6E). These observations indicate that ion fluxes through MCU are a required component of the mechanism that drives injury-induced mitochondrial fission *in vitro*. In order to determine whether mitochondrial ion fluxes through MCU could also have a role in the *in vivo* response of axonal mitochondrial to spinal cord injury, adult Sprague-Dawley rats were injected with mtDsRed/GFP/AAV into the somatomotor cortex 4 weeks prior to the severing of CST axons at the C4-C5 level. Immediately following injury, gel foam soaked in either vehicle or 10 mM RU360 was placed at the site of injury. The animals were euthanized either 2 h or 24 h following injury and mitochondrial size as a function of time after injury and distance from the site of injury was analyzed as previously described. Treatment with RU360 resulted in longer mitochondria after injury compared to time-matched controls (Figure 6F). However, in all cases, the length of mitochondria was also shorter from that of uninjured controls (Figure 6F). Animals treated with RU360 showed greatly attenuated fragmentation in the vicinity of the injury site (0–100 micron) at either 2 h or 24 h post-injury (red percentages and *p* values in Figure 6F), indicating that mitochondrial ion fluxes through MCU are also a required component of the mechanism driving injury-induced mitochondrial fission *in vivo*.

## Discussion

Dysfunctional mitochondria have consistently been associated with neuronal disease and injury (Reddy et al., 2011; Galloway et al., 2012; Reddy, 2014; Luo et al., 2015; Wu et al., 2016, 2017; Cherubini and Gines, 2017). In this study, we used both *in vitro* and *in vivo* models to investigate the response of axonal mitochondria to axonal injury. Mitochondria within CST axons undergo rapid fragmentation following severing. Mitochondrial fragmentation was persistent within retraction bulbs even after mitochondria in more proximal regions of the axon had recovered to normal length. Injury-induced mitochondrial shortening was also found in three different *in vitro* models of axonal injury. Drp1-mediated fission of mitochondria into fragments smaller than 0.5 μm is associated with neuronal malfunction and cell death in a variety of neurotoxic and disease states (Karbowski, 2010; Otera et al., 2013; Kasahara and Scorrano, 2014; Pradeep et al., 2014; Reddy, 2014; Balog et al., 2016; Bertholet et al., 2016; Galluzzi et al., 2016; Hu et al., 2017). The observed fragmentation of axonal mitochondria following injury could severely reduce mitochondrial bioenergetics and impair growth from the cut axon tip. This is in agreement with studies showing that, at the tissue level, mitochondrial respiration following spinal cord injury is impaired (Jin et al., 2004; McEwen et al., 2007; Sullivan et al., 2007; Patel et al., 2009, 2010), and there is a rise in mitochondrial oxidative stress (Jin et al., 2004; McEwen et al., 2007; Sullivan et al., 2007; Patel et al., 2009).

These findings raise the question of the physiological purpose for injury-induced mitochondrial fission. The speed and distance of mitochondrial movement are inversely related to mitochondrial length (Steketee et al., 2012; Narayanareddy et al., 2014). Additionally, mitochondrial fission is necessary for axonal branching (Spillane et al., 2013; Armijo-Weingart et al., 2019). This raises the possibility that the fission of mitochondria into smaller, but not dysfunctional, fragments could increase their transport rate within axons to aid the redistribution of healthy mitochondria within axons, increasing the growth potential. However, it is unclear why many axonal mitochondria within the CNS become fragmented following injury, rendering them incapable of aiding in neuronal growth and recovery.

Retraction bulbs are formed as a consequence of new growth cone formation failure and the continued flow of axoplasm to the injured tip resulting in a swollen, bulbous axonal end. Retraction bulbs are marked by the presence of accumulated organelles, such as smooth endoplasmic reticulum and mitochondria, as well as disorganized microtubules (Hill, 2017). The fragmentation of mitochondria following injury may contribute to the formation of retraction bulbs, a main contributor to the lack of regenerative potential in the CNS. We observed that fragmented mitochondria persist within retraction bulbs up to 2 weeks after injury (Figure 1E), a time at which axonal mitochondria have re-attained lengths comparable to axonal mitochondria in non-injured controls. For axonal regrowth to occur after injury, the injured axon must first form a growth cone, an energy demanding multistep process that relies on ATP produced by healthy mitochondria (Campbell and Holt, 2001; Verma et al., 2005; Hill, 2017). Multiple recent studies show that increasing delivery of healthy mitochondria into injured axon tips positively contributes to axonal regeneration (Cartoni et al., 2016; Han et al., 2016; Zhou et al., 2016). Therefore, we posit that the injury-induced mitochondrial fragmentation in the CNS results in energy deficiency at the cut axon tip, leading to the formation of a retraction bulb and the failure of growth cone formation. Our observation that inhibiting injury-induced mitochondrial fragmentation using mDivi-1 reduced the number of retraction bulbs reinforces this hypothesis. Additionally, inhibiting injury-induced mitochondrial fragmentation reduced the lesion volume. It should be noted that the reduction in lesion volume and the reduction in the number of retraction bulbs may be caused by independent effects of mDivi-1 in different cell types. A previous study demonstrated that mDivi-1 treatment following spinal cord injury prevented astrocyte activation and astroglial scar formation, thereby reducing lesion volume (Li et al., 2016). Nonetheless, our findings demonstrate that inhibition of injury-induced mitochondrial fragmentation prevents retraction bulb formation and may ultimately contribute to axonal regeneration following injury.

Dysfunctional mitochondria are detrimental due to their incapability to produce enough ATP to meet cellular demand and increased generation of reactive oxygen species (ROS). Dysfunctional mitochondria are removed through mitophagy. Following spinal cord injury, a sharp increase in mitophagy occurs, as measured by LC3-mitochondria colocalization, beginning at 2 h after injury and peaking at 1 day following injury, before returning to non-injury control levels 7 days after injury. This timeline coincides with the observation that mitochondria in the vicinity of the injury site are fragmented up to 3 days following injury before showing partial recovery of normal mitochondrial length. These observations are consistent with the notion that the injury-induced mitochondrial fragments are dysfunctional and must therefore be removed from the axon in the interest of maintaining neuronal health (Ribas and Lingor, 2015; Ribas et al., 2015). Activation of autophagy using a cell permeable Tat-Beclin1 peptide, a key component of autophagosome formation (Molino et al., 2017), reduces retraction bulb formation acutely, as well as axonal dieback 6 weeks after SCI (He et al., 2016). In contrast, inhibiting VPS34, a phosphoinositide-3-kinase (PI3K) involved in autophagosome initiation, using 3MA prevents acute axonal degeneration and neuronal degeneration after either optic nerve injury or SCI (Knoferle et al., 2010; Bisicchia et al., 2017). Although the underlying models are different and could differentially modulate the rate at which autophagosomes are cleared, another explanation is that 3MA is a general PI3K inhibitor, and not specific to VPS34 (Knight and Shokat, 2007; Kong and Yamori, 2008). Our observations suggest that the specific promotion of mitophagy following spinal cord injury possibly proves beneficial in axonal regeneration and recovery.

Calcium dynamics following injury are important in determining whether a new growth cone or a retraction bulb will form (Bradke et al., 2012), thus we investigated whether calcium may contribute to Drp1-dependent injury-induced mitochondrial fragmentation. Following injury, there was an increase in both cytosolic and mitochondrial calcium content. Increases in mitochondrial calcium are due to increased permeability of the MCU to divalent ions, largely calcium and zinc. The increase in mitochondrial calcium content was not blocked by the application of mDivi-1, indicating that the observed mitochondrial uptake is independent of injury-induced mitochondrial fission. Inhibiting ionic fluxes through the MCU using RU360, both *in vitro* and *in vivo*, blocked injury-induced mitochondrial fission, showing that mitochondrial ionic uptake is a component of the mechanism underlying injury-induced mitochondrial fragmentation. Mitochondria are composed of an outer mitochondrial membrane (OMM) and an inner mitochondrial membrane (IMM), both of which must be fully constricted for fission to occur (Kuroiwa et al., 2008). Constriction of the IMM was shown to be dependent on mitochondrial calcium uptake, and could be blocked by administration of RU360 (Cho et al., 2017). Thus, the influx of mitochondrial calcium following injury may be required for fission to come to completion due to its role in IMM constriction. Additionally, Drp1 alone is unable to initiate mitochondrial constriction because Drp1 oligomers can only form contractile rings with a diameter of up to 100 nm, while mitochondria have diameters between 0.5 and 1.0 μm (Ingerman et al., 2005; Mears et al., 2011). Therefore, mitochondrial fission requires a pre-constriction step, in which actin filaments promote direct contact of mitochondria with the endoplasmic reticulum (ER; Friedman et al., 2011; Korobova et al., 2013). A rise in cytosolic calcium promotes actin-dependent pre-constriction, bringing mitochondria into closer contact with ER, allowing for efficient uptake of calcium into mitochondria through the MCU (Chakrabarti et al., 2018). These results suggest that following injury, a large influx of calcium into the cytosol results in actin polymerization, which drives mitochondrial pre-constriction and mitochondrial-ER contact. This in turn leads to an uptake of calcium into the mitochondria through the MCU and promotes constriction of the IMM priming mitochondria for Drp1-dependent fission. We cannot rule out a possible role for ionic fluxes through the MCU in regulating aspects of the Drp1-dependent mechanism of fission following injury, but given that the latter is occurring on the outer mitochondrial membrane while the fluxes are inside the mitochondrion this seems unlikely. While a case can be made for the role of mitochondrial calcium uptake, zinc uptake may also contribute. During fission, Drp1 promotes the flux of zinc through the MCU that in turn serves to suppress mitochondrial membrane potential and target mitochondria for mitophagy (Cho et al., 2019). Thus, both calcium and zinc fluxes through the MCU could contribute to injury-induced mitochondria fragmentation and mitophagy. Regardless of the specific ion responsible, the mechanistic insight that fluxes through MCU is required may provide yet another target for inhibiting injury-induced mitochondrial fragmentation and perhaps promoting axonal regeneration in the future.

This report details the novel findings that following injury, axonal mitochondria of CST neurons undergo rapid fragmentation in a Drp1-dependent process. Mitochondrial fragments persist in retraction bulbs and may play a role in their initial formation or maintenance. An axonal injury also results in an increase in mitophagy, indicating that the injury-induced mitochondrial fragments are dysfunctional. Finally, we show that mitochondrial calcium uptake is a mechanistic contributor to injury-induced mitochondrial fragmentation. These observations pave the way for new strategies aimed at axonal mitochondrial dynamics in the effort to prevent axonal dieback, and to promote axonal regeneration following injury.

## Data Availability Statement

The raw data supporting the conclusions of this article will be made available by the authors, without undue reservation.

## Ethics Statement

The animal studies were reviewed and approved by Institutional Animal Care and Use Committee.

## Author Contributions

JK, SL, and AP performed the majority of experimental studies. JK, GG, and GS designed the majority of experiments, performed data analyses, wrote and edited the manuscript. All authors contributed to the article and approved the submitted version.

## Conflict of Interest

The authors declare that the research was conducted in the absence of any commercial or financial relationships that could be construed as a potential conflict of interest.

## Supplementary Material

SUPPLEMENTARY FIGURE 1 Distribution of mitochondrial length measurements for the post-injury time course and distance from injury suite analysis. Each dot represents a mitochondrion.
