# Supplementary figures and images for: Axotomy Induces Drp1-Dependent Fragmentation of Axonal Mitochondria
Source: Front Mol Neurosci. 2021 Jun 3;14:668670. doi: 10.3389/fnmol.2021.668670 (PMC8209475; doi:10.3389/fnmol.2021.668670)

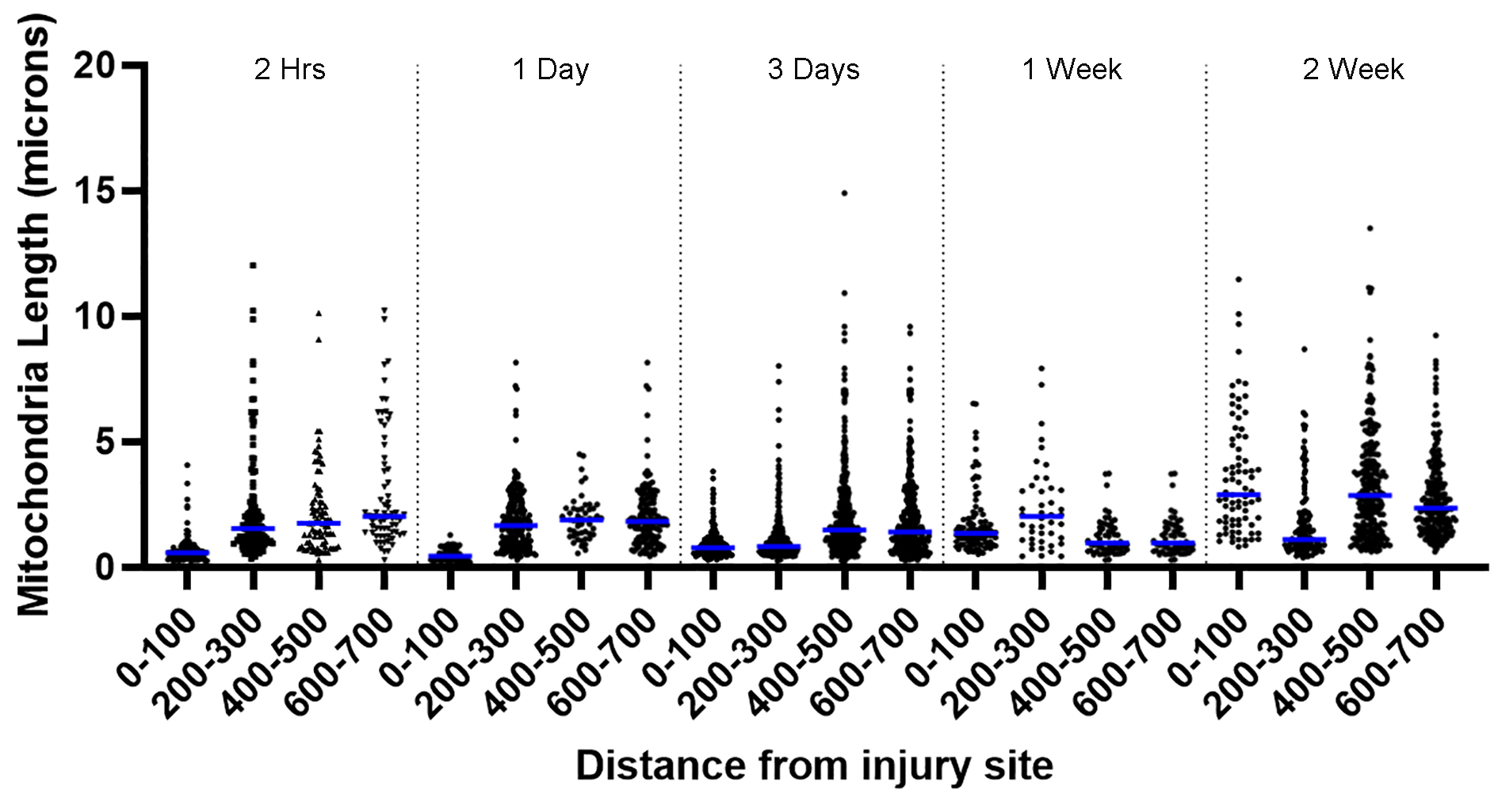

Supplement: SUPPLEMENTARY FIGURE 1 — Distribution of mitochondrial length measurements for the post-injury time course and distance from injury suite analysis. Each dot represents a mitochondrion. [file Image_1.JPEG]
